# Physicochemical and Biological Characterization of Gelatin/Alginate Scaffolds Reinforced with *β*-TCP, FDBA, and SrHA: Insights into Stem Cell Behavior and Osteogenic Differentiation

**DOI:** 10.1155/2024/1365080

**Published:** 2024-08-19

**Authors:** Sadra Mohaghegh, Hanieh Nokhbatolfoghahaei, Sahar Baniameri, Hekmat Farajpour, Massoumeh Jabbari Fakhr, Fatemeh Shokrolahi, Arash Khojasteh

**Affiliations:** ^1^Shahid Beheshti University of Medical Sciences, Tehran, Iran; ^2^Department of Artificial Intelligence, Smart University of Medical Sciences, Tehran, Iran; ^3^University of Tehran, Tehran, Iran; ^4^Iran Polymer and Petrochemical Institute, Tehran, Iran

## Abstract

Bone tissue engineering necessitates the development of scaffolds with optimal properties to provide a suitable microenvironment for cell adhesion, proliferation, and osteogenic differentiation. The selection of appropriate scaffold materials remains a critical challenge in this field. In this study, we aimed to address this challenge by evaluating and comparing the performance of hydrogel scaffolds reinforced with *β*-tricalcium phosphate (*β*-TCP), allograft, and a combination of allograft and strontium hydroxyapatite (SrHA). In this study, scaffolds containing the following compounds with a weight ratio of 75 : 25 : 50 were made using a 3D printer: group (1) alginate + gelatin + *β*-TCP (TCP), group (2) alginate + gelatin + allograft (Allo), and group (3) alginate + gelatin + allograft + strontium hydroxyapatite (Str). Stem cells extracted from rat bone marrow (rBMSCs) were cultured on scaffolds, and cell proliferation and differentiation tests were performed. Also, the physical and chemical properties of the scaffolds were investigated. The two/one-way analysis of variance (ANOVA) by Tukey's post hoc test was performed. There was no significant difference between scaffolds with pore size and porosity. TCP scaffolds' mechanical strength and degradation rate were significantly lower than the other two groups (*P* < 0.05). Also, the swelling ratio of Allo scaffolds was higher than in other samples. The amount of cell proliferation in the samples of the TCP group was lower than the other two, and the Allo samples had the best results in this concern (*P* < 0.01). However, the scaffolds containing strontium hydroxyapatite had significantly higher bone differentiation compared to the other two groups, and the lowest results were related to the scaffolds containing *β*-TCP. Hydrogel scaffolds reinforced with allograft or its combination with strontium showed better physicochemical and biological behavior compared to those reinforced with *β*-TCP. Besides, adding strontium had a limited impact on the physicochemical features of allograft-containing scaffolds while improving their potential to induce osteogenic differentiation.

## 1. Introduction

Considering the structural resemblance of the polymer-ceramic composite materials to natural bone, they have been considered interesting options for bone tissue engineering procedures. This can be justified by assuming that these materials enable researchers to optimize the features of the scaffolds by altering the weight ratio (wt %) of the mineral and organic phase of the scaffold. Besides, the products produced due to the degradation of the mineral phase can neutralize the acidic products made as a result of polymer degradation [1]. Finally, it has been shown that polymer-ceramic scaffolds can provide a more suitable platform for stem cell adhesion, proliferation, and osteogenic differentiation compared to pure usage of mineral or organic materials [2–5].

Due to its resemblance to the organic phase of the natural bone, gelatin can be a promising option for the polymeric phase of the scaffold [6]. However, this material has limited structural stability in the pure form, necessitating researchers to improve its features by adding other polymeric phases, such as alginate [7–9]. Low cost, accessibility, and biocompatibility make alginate a proper additional agent to gelatin [6, 10]. Although studies showed acceptable results for alginate-fabricated scaffolds, it has not been proven whether they are entirely degradable [11–13]. Indeed, alginate-gelatin composites form covalent and ionic bonds, providing a niche for cellular adhesion while maintaining integrity. However, tuning porosity, mechanics, viscosity, and other properties to support 3D structures and regeneration remain challenging due to uncontrolled swelling, degradation, and limited capacity. Continued research modifies compositions and fabrication techniques to enhance these hydrogels for regenerative applications [5].

Studies improved the biological features of scaffolds by adding different chemical ions, specifically divalent ions, which can significantly enhance the osteogenic differentiation of stem cells [3, 14, 15]. Strontium is a natural element that can replace the calcium ion in bone, increase osteoblast activity, and decrease osteoclast function [16–18]. Application of this ion has been shown for the treatment of osteoporosis, bone fracture, and bone regeneration [17, 19, 20]. It has acceptable biocompatibility and low toxicity, making it an appropriate option for clinical applications [21, 22].

The development of scaffold materials with optimized properties is fundamental to the progress of bone tissue engineering strategies. Although different compositions of the scaffolds have been analyzed previously, the majority of them focused on synthetic materials such as PCL as the organic phase and *β*-tricalcium phosphate or other calcium phosphate-derived material for the ceramic phase [23–27]. In this context, our research introduces a novel approach by combining alginate, allograft, and strontium hydroxyapatite (SrHA) within a 3D-printed scaffold, which has not been extensively investigated before. In the current study, we intended to improve the physicochemical and biological characteristics of the composite scaffolds made of natural osteo-inducing materials (i.e., gelatin and allograft). Integrating these materials into the scaffold structure through 3D-printing techniques offers precise control over the spatial arrangement of the biomaterial, thereby enhancing the scaffold's osteogenic potential. Consequently, this innovative combination holds considerable potential as a viable strategy for advancing bone tissue engineering applications.

We hypothesized that supplementing our gelatin/alginate hydrogel scaffolds with freeze-dried bone allograft (FDBA) would enhance osteogenic differentiation and improve scaffold properties versus *β*-tricalcium phosphate (*β*-TCP). The results provide insights into optimizing these scaffolds. FDBA and its composite with strontium hydroxyapatite (SrHA) demonstrated enhanced osteogenic differentiation over *β*-TCP without impairing proliferation. FDBA also improved degradation, swelling, and mechanical strength compared to *β*-TCP. Thus, FDBA and FDBA/SrHA supported our hypothesis and show potential as scaffolds to guide robust osteogenic differentiation through optimized characteristics versus *β*-TCP. These findings offer guidance on developing osteoinductive hydrogel scaffolds and validate our hypothesis that FDBA incorporation improves critical scaffold qualities for bone regeneration.

## 2. Materials and Methods

### 2.1. Materials

Medical-grade freeze-dried bone allograft nanoparticles with an average size of 42 *µ*m (FDBA) (TRC, Tehran, Iran), gelatin (Gel) (Sigma-Aldrich, St. Louis, MO, USA), sodium alginate (Alg) (Sigma-Aldrich, USA), Dulbecco's modified eagle medium, (DMEM) (Thermo Fisher Scientific, USA), Dulbecco's phosphate buffered saline (PBS) (Sigma-Aldrich, USA), fetal bovine serum (FBS) (Thermo Fisher Scientific, USA), trypsin-ethylenediaminetetraacetic acid (Thermo Fisher Scientific, USA), ascorbate-2 phosphate (Sigma-Aldrich, USA), penicillin/streptomycin (Thermo Fisher Scientific, USA), indomethacin (Sigma-Aldrich, USA), CD44-FITC (BioLegend, USA), CD105-PE (eBioscience, USA), CD45-FITC (BioLegend, USA), CD34-PE (BioLegend), 4% paraformaldehyde (Sigma-Aldrich, USA), Alizarin Red S (Bio Idea, Iran) or Oil Red O (Bio Idea, Iran), 3-(4, 5-dimethylthiazol-2-yl)-2, 5-diphenyltetrazolium bromide (MTT, Sigma-Aldrich, USA), dimethyl sulfoxide (DMSO) (Sigma-Aldrich, USA), 40, 6-diamidino-2-phenylindole (DAPI) (Sigma-Aldrich, St. Louis, MO, USA), alkaline phosphatase (ALP) kit (Abcam, USA), BCA Quantification kit (DNAbiotech, Tehran, Iran), and TRIzol® (DNAbiotech, Tehran, Iran) reagent were purchased for this study.

The osteogenic medium used in this study consisted of low glucose DMEM, 10% FBS, 50 *μ*g/ml ascorbate-2 phosphate, 10^−8^ M dexamethasone, 10 mM *β*-glycerophosphate, and 1% penicillin/streptomycin. The adipogenic medium consisted of low glucose DMEM, 10% FBS, indomethacin 100 nM, dexamethasone 50 *μ*M, insulin 1 *μ*M, and 1% penicillin/streptomycin.

Distilled water was produced using a double water distiller system (Mobin Teb, Iran). *β*-TCP powder was manufactured according to a previously mentioned protocol [14, 28] with an average particle size of 45 *μ*. All other chemicals and reagents were analytical and used as received without any further purification.

### 2.2. Preparation of SrHA Nanocrystals

Synthesis of Sr-doped HAp was performed by precipitation with increasing ion exchange between calcium (Ca) and Sr, similar to a previous report with slight modifications [29, 30]. Sr-doped HA nanoparticles (Sr-HAp) were produced according to the literature [29]. In brief, to a Sr2+ containing 5 wt % solution of Ca (NO_3_)_2_·4H_2_O (1 M), in DD water, diammonium hydrogen phosphate (0.6 M) was added at room temperature, while pH was carefully adjusted at 11 over the addition process. The solution was stirred for 24 hours while Sr-HAp precipitated out. The nanoparticles were washed with DD water, centrifuged in triplicate, rinsed, and freeze-dried. Sr-HAp was stored in a desiccator before use. To characterize the nanocrystals, scanning electron microscopy (SEM), Fourier transform infrared spectroscopy (FTIR), and energy-dispersive X-ray analysis (EDXA) analyses were performed according to Ghorbani et al. [29].

### 2.3. Scaffold Fabrication and Cross-Linking

Gelatin, sodium alginate, freeze-dried bone allograft, *β*-tricalcium phosphate, and strontium hydroxyapatite were sterilized with UV lamp irradiation for 30 minutes. Gelatin, alginate, and bioceramic were dissolved in distilled water at 40°C for 4 hours to prepare printing blends. The bioceramic amount equaled the sum of alginate and gelatin. SrHA-containing samples had 75% of the bioceramic content as allograft and the remaining 25% as SrHA as shown in Table 1. Scaffolds were manufactured using an extrusion-based 3D printer (Sizan Group, Kashan, Iran) at room temperature with a nozzle size of 450 *µ*m and speed of 10 mm/s. The 3D scaffold had a cubical form with a base side of 4 × 4 mm, height of 3 mm, a strut size of 450 *μ*m, pore size of 450 *μ*m, and porosity of 50%. Cross-linking of the scaffolds was performed with either 10% CaCl2 for 10 minutes or genipin. A pilot study was conducted to determine the appropriate genipin solvent and cross-linking duration, evaluating cell proliferation with Alamar Blue staining. [31].

### 2.4. X-Ray Diffraction and Fourier Transform Infrared Spectroscopy (FTIR) Analysis

The scaffolds' phase was characterized using wide-angle X-ray diffraction ((XRD) D/Max 2550 V; Rigaku, Japan). Scanning was performed in the range of diffraction angle 2*θ* = 20°–60° at a rate of 2 min^−1^ with a step width of 0.02°2*θ*. Cu K*α*1 radiation at 40 kV and 40 mA current strength were employed to identify the crystal peak of the mineral phase. The average crystal dimensions of FDBA, *β*-TCP, and SrHA were calculated using Scherrer's equation, where *D* is the average crystal diameter in the direction that is perpendicular to the lattice planes, hkl is the Miller indices of the analyzed planes, *K* is the crystallite-shape factor (*K* 1/4 0.94), *l* is the wavelength of the X-ray (∼1.540598 Å Cu-Ka radiation), *b* is the line broadening full width at half the maximum intensity (FWHM) in radians, and *q* is the Bragg diffraction angle.

The Bragg peaks used for Scherrer's equation were 112 for FDBA, 211 for SrHA, and 217 for *β*-TCP.

The chemical composition of the scaffolds was investigated using Fourier transform infrared spectroscopy (FTIR; Spectrum GX, USA). Each sample, along with KBr, was ground in an agate mortar at a ratio of about 1 : 20 and compressed into tablets. The spectral range was set from 4000 to 400 cm^−1^ with a resolution of 4 cm^−1^ and a scan time of approximately 100 seconds. The equation is as follows:

(1) $$\begin{array}{c} D_{\text{hkl}}=K\frac{\lambda }{\beta_{\text{hkl}}}\cos\;\theta . \end{array}$$

### 2.5. Porosity and Morphology of Scaffolds

Scanning electron microscopy (SEM, Hitachi SU3500, Japan) was used to analyze the surface morphology of the scaffold. After lyophilizing, all samples were precoated with a conductive layer of sputtered gold (10 nm). The micrographs were obtained at 15 kV of accelerating voltage. The pore size and strut thickness were analyzed using ImageJ software (NIH, USA). Mean pore and strut size were calculated using image analysis SEM samples.

### 2.6. Mechanical Properties of Composite Scaffolds with Different Compositions

To evaluate the compressive properties of the scaffolds (4 × 4 × 3 mm) in a wet condition, a universal testing instrument (SANTAM, Iran) with 100 N load was used in the compressive mode at 37°C. The compression rate was set at 0.5 mm s^−1^. The compressive modulus was subsequently calculated from the slope in the linear region corresponding to 10–25% strain. Five measurements were performed for each hydrogel condition, and values were reported as mean ± standard deviation (SD).

### 2.7. *In Vitro* Degradation and Swelling of the Scaffolds

To evaluate degradation, freeze-dried samples were weighed (*ω*0) and immersed in distilled water (water-scaffold ratio of 312.5 ml/g) at 37°C with agitation. Samples were retrieved at various time points (1, 7, 14, 21, 28, and 56 days), freeze-dried, and reweighed (*ω*1). Degradation was calculated as (*ω*1–*ω*0)/*ω*0.

To analyze swelling, scaffolds were lyophilized and weighed (*ω*0). They were then immersed in distilled water at 37°C for 24 hours. The swollen weight was recorded (*ω*1), and the swelling ratio was calculated as (*ω*1–*ω*0)/*ω*0 [3].

### 2.8. Stem Cell Isolation and Characterization

Two male 2-week-old Sprague–Dawley rats were euthanized with CO2 asphyxiation (flow rate of 2 L/min until breathing movements ceased for 1 min) for stem cell isolation. Rat femur and tibiae bone were dissected under sterile conditions and kept in DMEM at a low temperature according to a previously reported protocol [14]. The research protocol is according to ARRIVE guidelines and approved by the Ethical Committee of the dental school, Shahid Beheshti University of Medical Sciences (ethical code: IR.SBMU.DRC.REC.1401.004). The bones were washed three times with PBS and 5X Pen-Strep. Bone heads were removed, and marrow was flushed out with DMEM injection. The obtained substrate was centrifuged, and the sediment was resuspended in DMEM. Plates were then incubated in the standard medium at 37°C and 5% CO2.

Flow cytometry was performed to analyze the expression of markers specific to mesenchymal stem cells (MSC-specific). In detail, passage three undifferentiated mesenchymal stem cells were harvested using 0.05% trypsin-EDTA, resuspended in PBS at a concentration of 10^5^ per sample, and stained with the FITC-conjugated monoclonal antibodies listed below: CD44-FITC, CD105-PE, CD45-FITC, and CD34-PE. Following incubation, cells were cleaned with PBS and analyzed using the FACSCalibur (BD Biosciences, USA) and FlowJo 7.6.1 software (BD, USA).

Their ability to differentiate into osteoblasts and adipocytes was analyzed to evaluate the multidifferentiation potential of stem cells. For this purpose, 25 × 10^3^ cells (passage 3) were grown in either osteogenic or adipogenic medium as previously described [3]. To analyze the osteogenic and adipogenic differentiation potential, cells were incubated for 14 and 21 days, respectively.

Next, for staining, the samples were cleaned twice with PBS before being fixed at room temperature with 4% paraformaldehyde. After being rinsed with distilled water, the cells were stained with Alizarin Red S or Oil Red O. Inverted light microscopy images of the labeled cells were used to assess the mineral deposition and adipogenesis. Images of the cells cultured in a standard medium for four days were also taken as the control.

### 2.9. Cell Adhesion and Proliferation

A total of 2.5 × 10^4^ passage three rBMSCs were seeded on each scaffold. Scaffolds were then placed in standard medium (i.e., DMEM high glucose+ 15% FBS + 1% antibiotics (100 U/ml penicillin and 100 mg/ml streptomycin sulfate)) and incubated at 37°C and 5% CO2. The culture medium was refreshed every other day.

Cell attachment and morphology on scaffolds were analyzed using surface electron microscopy after five days of incubation. The samples were fixed with 4% paraformaldehyde for 45 minutes, rinsed with PBS, and dehydrated using descending ethanol solutions (25%, 50%, 70%, 80%, 90%, and 100% gradient concentrations, 10 minutes each). A 10 nm gold layer was sputter-coated onto the samples, which were then scanned using scanning electron microscopy (SEM, Hitachi SU3500, Japan).

On the 5^th^ day, DAPI (4,6-diamidino-2-phenylindole) staining was performed based on the manufacturer's instruction to visualize live cells. In brief, scaffolds were rinsed with PBS three times, fixed with 4% paraformaldehyde for 45 min, and rinsed again with PBS. Next, DAPI stain solution was added to the fixed samples and incubated for 2 min. Samples were again rinsed with PBS three times. A fluorescence microscope was used to analyze cells.

rBMSCs proliferation was evaluated using the 3-(4, 5-dimethylthiazol-2-yl)-2, 5-diphenyltetrazolium bromide (MTT) assay on days 1, 3, and 5. The samples were rinsed with PBS three times and then incubated with 10% MTT solution in DMEM for 4 hours. Afterwards, 200 *μ*l of dimethyl sulfoxide (DMSO) was added and vortexed for 30 seconds. The samples were analyzed using an ELISA reader set at 570 nm (BioTek, USA).

Alamar Blue staining was also performed to evaluate stem cell proliferation further. In brief, the standard medium was exchanged with medium containing 10% Alamar Blue and incubated for 4 h. Next, Alamar Blue fluorescence was measured at 530/590 nm using an ELIZA reader (BioTek, Winooski, VT, USA).

### 2.10. Cell Differentiation

#### 2.10.1. Alkaline Phosphatase (ALP) Activity and Total Protein Count

The ALP activity was evaluated using an alkaline phosphatase (ALP) kit as previously described [14]. Cell-seeded scaffolds were incubated in the osteogenic medium for 1, 7, and 14 days. At each time point, the scaffolds were removed, rinsed with PBS, and mixed with cell lysis solution. After undergoing freeze-thaw cycles, the supernatant was obtained by centrifugation. A portion of the solution was mixed with p-nitrophenyl phosphate substrate, and the absorbance was measured at 405 nm using an ELISA reader. The assay buffer was prepared, and the reaction was stopped with NaOH. The total protein amount was determined using a BCA Protein Assay kit.

#### 2.10.2. Analysis of Gene Expression

Total RNA was extracted after 7 and 14 days, using TRIzol® reagent according to the manufacturer's instruction and stored at −80°C. A cDNA synthesis kit was used in a thermocycler GeneAmp® PCR System 9700 PE (Applied Biosystems, Foster City, CA, USA) for cDNA synthesis according to manufacturer instructions. Real-time PCR reactions were performed using 1 *µ*L of 5x diluted cDNA and SYBR® Green Master Mix in a LightCycler® (Roche Diagnostics). The procedure for all genes was performed in the following steps: 10 min preincubation at 95°C, followed by 45 cycles of amplification at 95°C for 2 s, 56°C for 8 s, 72°C for 10 s, and 82°C for 5 s, after which melting curve analysis was performed. In each run, the reaction mixture without cDNA was used as the negative control. Utilized primers (Life Technologies^TM^) were as follows:

 
*BMP-2*: (forward: 5′-gac tgc ggt ctc cta aag gtc-3′, reverse: 5′-gga agc agc aac gct aga ag-3′)
 
*Runx2*: (forward: 5′-gaa ccc aga agg cac aga ca-3′, reverse: 5′-act tgg tgc aga gtt cag gg-3′)
 
*GAPDH*: (forward: 5′-gac ttc aac agc aac tcc cac, reverse: 5′-tcc acc acc ctg ttg ctg ta-3′)

Relative quantification was performed using the comparative CT method (also known as the 2^−ΔΔCt^ method) [32], and results were reported relative to the calibrator group (samples of day 1 of each study group). All reactions were performed in duplicates, and each study group's three samples were considered for analysis.

### 2.11. Statistical Analyses

Prism 8.3.0 (GraphPad Software Inc., San Diego, CA, USA) was used for data analysis. Data were expressed as mean ± SD. Two/one-way analysis of variance (ANOVA) by Tukey's post hoc test was performed. A *P* value of less than 0.05 was considered statistically significant.

## 3. Results

### 3.1. SrHA Characterization

**Figure 1:**
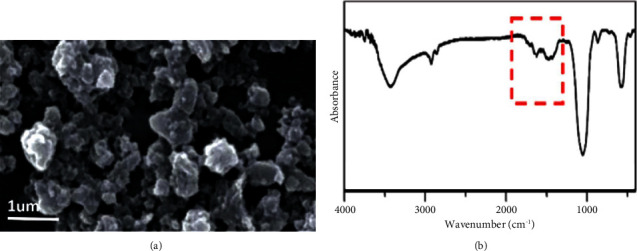
(a) SEM image of Sr-doped HA particles and (b) FTIR spectrum of the particles demonstrating characteristic vibration bands at 1623 cm^−1^ (OH stretching), 1018 and 567 cm^−1^ (PO_4_^−3^), and 1623 and 869 cm^−1^ (CO_3_^−2^), indicating the formation of the apatite structure. The (Sr + Ca)/*P* ratio of approximately 1.67 observed in the EDXA analysis confirms the successful synthesis of SrHA, comparable to the Ca/*P* ratio in natural HA.

The synthesized bioceramic nanoparticles (SrHA) were characterized by SEM, FTIR, and energy-dispersive X-ray analysis (EDXA).  Figure 1(a) shows the SEM image of SrHA synthesized in this study, and Figure 1(b) shows the FTIR trace of the Sr-doped HA particles.

In FTIR trace of the SrHA particles (Figure 1(b)), the vibration bands appearing at 1623 (OH stretching), 1018 and 567 (PO_4_^−3^), and 1623 and 869 (CO_3_^−2^) cm^−1^ prove the formation of the apatite structure [30].

EDXA analyses of the Sr-doped HA showed a (Sr + Ca)/P of about 1.67 which is close to that of Ca/P in natural HA and proves that the synthesis resulted in the formation of SrHA.

### 3.2. Scaffold Fabrication

**Figure 2:**
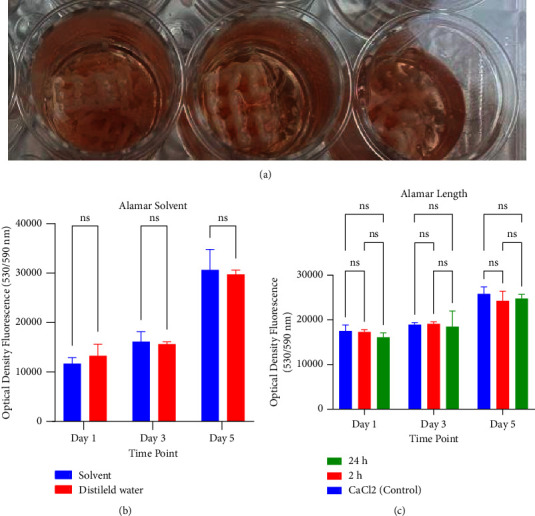
(a) Scaffold collapse after 14 days in the case of being cross-linked with CaCl_2_ 10% for 10 minutes. (b) A comparison of cell viability in the case of using genipin solvent or distilled water for cross-linking showed no significant differences between these two protocols. (c) A comparison of cell viability in the case of using genipin + solvent or CalCl_2_ for cross-linking the scaffolds for 2 hours or 24 hours showed no significant differences between these two protocols (ns: not significant). The comparison was statistically analyzed with a two/one-way analysis of variance (ANOVA).

Scaffolds cross-linked with 10% CaCl_2_ for 10 minutes collapsed after 14 days (Figure 2(a)). Thus, CaCl_2_ was not considered for cross-linking. Regarding genipin, the Alamar Blue analyses showed that the optimal protocol for cross-linking, which has minimal impact on cell viability, was using genipin dissolved in its solvent and exposing the scaffolds to genipin for 24 h (Figures 2(b) and 2(c)).

**Figure 3:**
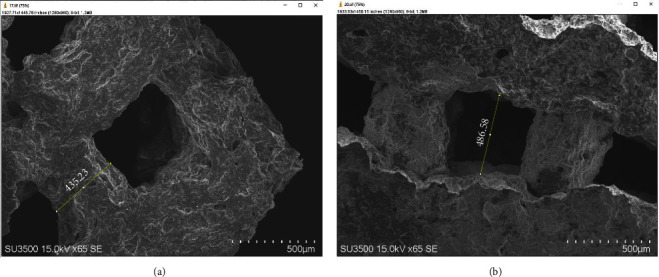
SEM images of strut (a) and pore (b) size measurements. There were no significant differences identified in terms of the pore size and strut size of the scaffolds.

SEM analyses showed insignificant differences between the scaffolds' pore size and strut size with the designed pattern (Figure 3).

### 3.3. XRD and FTIR

**Figure 4:**
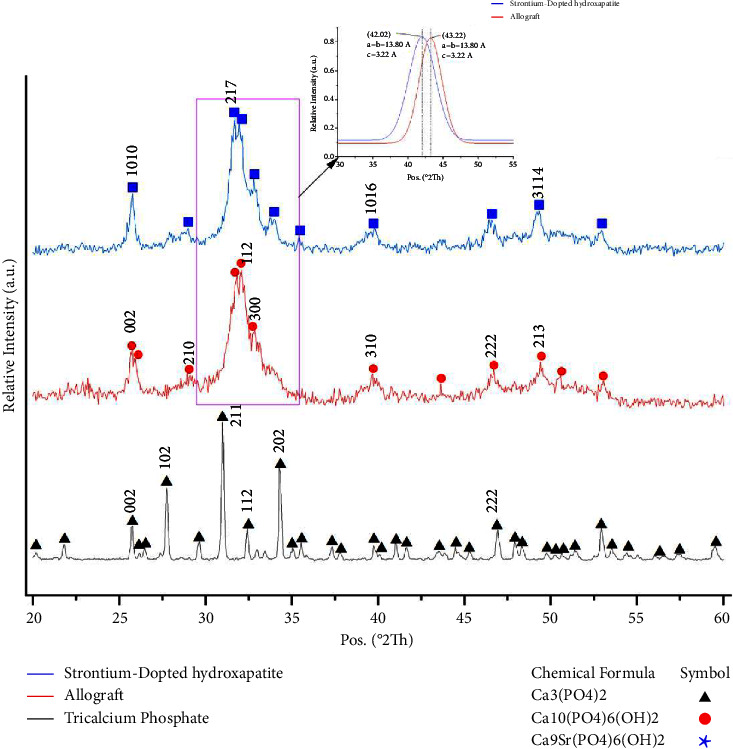
XRD spectra of the analyzed samples. XRD analysis verified the crystallographic properties of the materials, with characteristic peaks of FDBA, *β*-TCP, and SrHA matching the International Center for Diffraction Data (ICDD). The addition of SrHA resulted in a discernible difference between the Allo group and the Str group, as observed in the XRD spectra.

XRD further confirmed the crystallographic properties of the materials. The XRD spectra are shown in Figure 4. Characteristic peaks of FDBA, *β*-TCP, and SrHA were according to the International Center for Diffraction Data (ICDD). The difference between the Allo group and Str group due to the addition of SrHA is shown in Figure 4. The mean crystallite size was measured with Scherrer's equations in nanometers. The mean crystallite size measured for FDBA to the (112) Bragg peak was 13 nm and for *β*-TCP to (217) Bragg peak was 77 nm and for SrHA to (211) Bragg peak was 275 nm.

**Figure 5:**
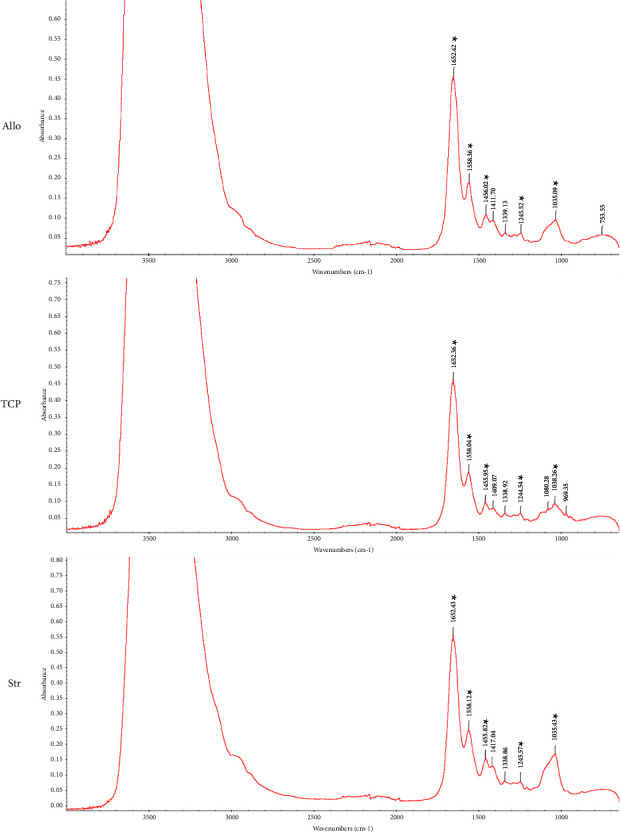
FTIR pattern of the analyzed samples. FTIR analysis showed characteristic peaks corresponding to specific functional groups in the different groups (Str, Allo, and TCP). These included the hydroxyl bond (OH) of sodium alginate, the antisymmetric stretch of COC, sodium alginate symmetry-COO stretching, and gelatin-related peaks. The observed patterns were consistent with the gelatin and sodium alginate spectra.

As shown in Figure 5, 3483 cm^−1^ (Str group), 3477 cm^−1^ (Allo group), and 3474 cm^−1^ (TCP group) are the sodium alginate hydroxyl bond (OH). Besides, 1038 cm^−1^ (TCP group) and 1035 cm^−1^ (Allo and Str groups) were the antisymmetric stretch of COC from sodium alginate. In addition, 1455 (Str and TCP groups) and 1456 (Allo group) were the sodium alginate symmetry-COO stretching (COOH group). About gelatin, 1652 (Allo, Str, and TCP groups) is the C=O group of amide I stretching of gelatin. Moreover, 1558 (Str, Allo, and TCP groups) is the NH group bending vibration of gelatin's amide II. Finally, 1244 (TCP group) and 1245 (Allo and Str groups) were the NH group of amide III of gelatin. All groups showed patterns consistent with the peaks of the gelatin and sodium alginate spectra.

### 3.4. Mechanical Strength and Swelling

**Figure 6:**
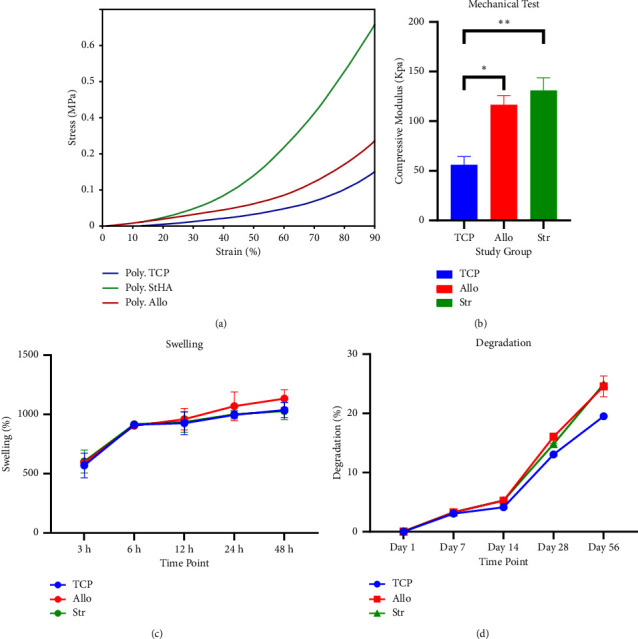
(a) Stress-strain curve of the samples. (b) A comparison of the compressive modulus of the study groups showed significantly higher values for Allo and Str samples than TCP samples (^*∗*^*P* = 0.02 and ^*∗∗*^*P* = 0.01), and the comparison was statistically analyzed with two/one-way analysis of variance (ANOVA). (c) Swelling percentage of the scaffolds showed increasing values for all study groups over time. (d) The same increasing pattern was seen for the degradation percentage of the samples.

Stress-strain curve and the calculated compressive modulus are shown in Figures 6(a) and 6(b). Samples of the TCP group had significantly lower compressive modulus compared to Allo (TCP: 56 ± 8.48 vs. Allo: 116.5 ± 9.19 kPa; *P* = 0.02) and Str (Str:131 ± 12.72 kPa; *P* = 0.01) groups. Besides, Str samples showed higher mechanical strength than Allo samples with insignificant differences (*P* = 0.44). As shown in Figure 6(c), scaffold swelling increased with time, and Allo samples had the highest level at each time point after 48 h. However, there was no significant difference in the swelling ratio of the study group after 48 h (TCP: 1036.41 ± 63.93%, Allo: 1132.64 ± 75.97, and Str: 1028.66 ± 72.55; *P* > 0.05).

### 3.5. Scaffold Degradation

The degradation percentage of the scaffolds is shown in Figure 6(d). The degradation rate increased over time, and after 56 days, TCP samples had the lowest degradation compared to Str and Allo samples (TCP: 19.5 ± 0.14%, Allo: 24.56 ± 1.75%, and Str: 24.95 ± 0.07%; *P* < 0.01), while there was no significant difference in the degradation percentage of Allo and Str samples (*P* = 0.69).

### 3.6. Stem Cell Characterization

**Figure 7:**
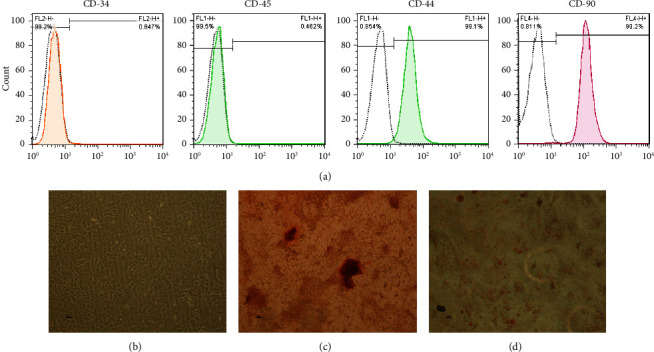
(a) Flow cytometric characterization of human rBMSCs; negative expression of CD34 (0.84%) and CD45 (0.46%) and positive expression of CD44 (99.1%) and CD90 (99.2%). (b) rBMSCs after 4 days in standard medium. (c) Alizarin Red S staining of MSCs in 2D culture 14 days after cell culturing in osteogenic medium. (d) Oil Red O staining of MSCs in 2D culture 14 days after cell culturing in adipogenic medium.

The flow cytometry results showed that the isolated rBMSCs expressed CD105 and CD44 but were consistently negative for CD34 and CD45 (Figure 7(a)). The osteogenic and adipogenic differentiation capacity of rBMSCs was confirmed by Alizarin Red S and Oil Red O staining (Figure 7(b)).

### 3.7. Stem Cell Adhesion and Proliferation

**Figure 8:**
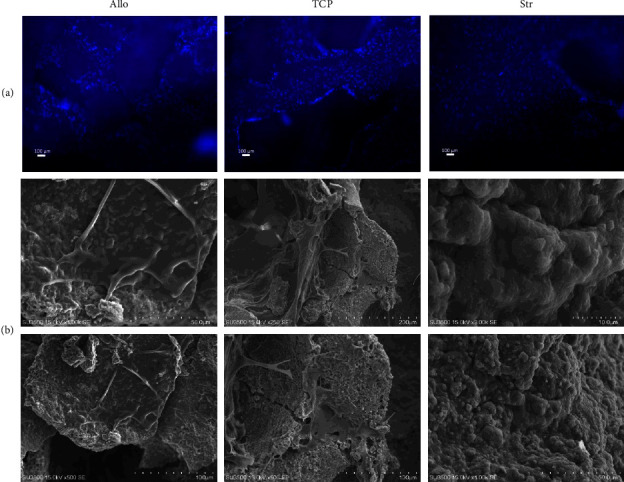
(a) DAPI (scale bar: 100 *µ*m) results showing proper cell adhesion and pseudopod formation in all study groups. (b) SEM DAPI (scale bar: 100 *µ*m) results showing proper cell adhesion and pseudopod formation in all study groups.

**Figure 9:**
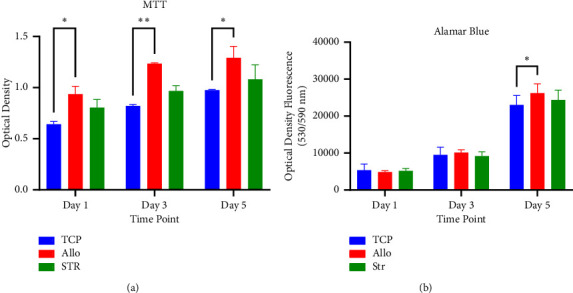
Cell proliferation analyses based on (a) MTT assay (^*∗*^*P* = 0.03 and ^*∗∗*^*P* = 0.005) and (b) Alamar Blue staining (^*∗*^*P* = 0.02). Results showed significantly higher cell proliferation in Allo samples compared to other groups. The comparison underwent statistical analysis using two-way analysis of variance (ANOVA).

Figure 8(a) shows the SEM images of the scaffolds after 5 days of stem cell cultivation. Stem cell bodies and pseudopods indicate proper adhesion of cells to the scaffolds. The surface roughness caused by bioceramics which aid stem cell adhesion is visible in the images. The results of DAPI staining are shown in Figure 8(b). Accordingly, scaffolds provided a good surface for cell adhesion and were adequately distributed on the scaffolds. Stem cell proliferation was analyzed with MTT and Alamar Blue analyses, and they both showed that these cell numbers increased from day 1 to 5 in all samples, and after 5 days, Allo samples had higher proliferation levels compared to TCP (MTT: *P* = 0.03 and Alamar Blue: *P* = 0.02) (Figures 9(a) and 9(b)). Both analyses showed no significant difference between the proliferation rate of Str and Allo samples (MTT: *P* = 0.21 and Alamar Blue: 0.16), while Str samples showed lower values.

### 3.8. Osteogenic Differentiation

#### 3.8.1. ALP Activity

**Figure 10:**
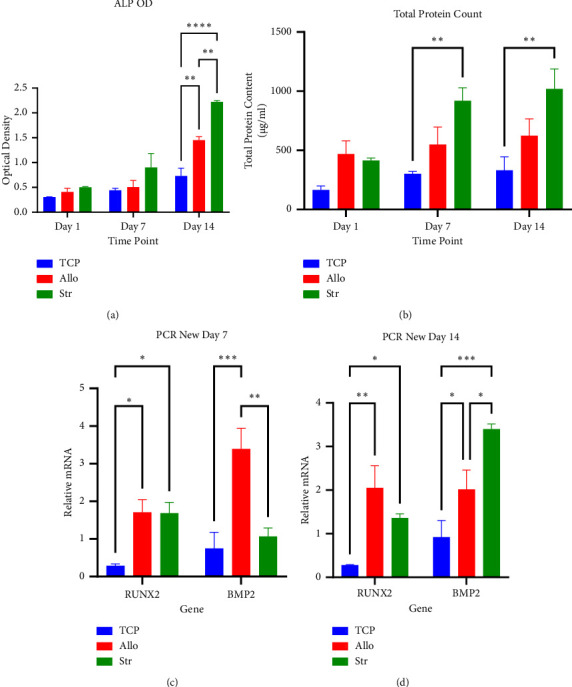
(a) ALP activity analyses showed significantly higher osteogenic differentiation of rBMSCs seeded on Str scaffolds compared to other samples (^*∗∗*^*P* = 0.004 and ^*∗∗∗∗*^*P* < 0.0001). (b) For protein count assay, results showed significantly higher values for Str samples (^*∗∗*^*P* = 0.002). ((c) and (d)) Results of gene expression analyses confirmed ALP activity results and showed higher osteogenic gene expression in Str samples after seven days (^*∗*^*P* = 0.01, ^*∗∗*^*P* = 0.001, and ^*∗∗∗*^*P* = 0.0007) and 14 days (^*∗*^*P* < 0.05, ^*∗∗*^*P* < 0.01, and ^*∗∗∗*^*P* = *P* < 0.001). All the comparisons were subjected to statistical analysis using two-way analysis of variance (ANOVA).

A comparison of ALP activity between the study groups is shown in Figure 10(a). Results showed that the ALP activity of all study groups increased from day 1 to 14. On day 14, Str samples had the highest activity with a significant difference between Allo and TCP groups (TCP: 0.73 ± 0.15, Allo: 1.45 ± 0.07, and Str: 2.22 ± 0.02; *P* < 0.01).

#### 3.8.2. Total Protein Count

This variable can indicate external cellular matrix production by stem cells, which is considered a sign of differentiation. The protein amount increased from day 1 to 14. After 7 and 14 days, Str samples had significantly higher values than Allo and TCP (*P* < 0.01). However, there was no significant difference between Allo with TCP (TCP: 331.08 ± 112.86 *µ*g/ml and Allo: 623.71 ± 142.12 *µ*g/ml; *P* = 0.27) and Str (Str: 1019.78 ± 168.61 *µ*g/ml; *P* = 0.08) groups (Figure 10(b)).

#### 3.8.3. RT-PCR Results

As shown in Figures 10(c) and 10(d), after 14 days, there was no significant difference in the *Runx2* expression of Str and Allo samples, although the latter showed higher values. However, the expression of *Runx2* was significantly lower in the TCP group compared to Allo (*P* < 0.01) and Str. Regarding BMP-2 expression, Str samples had significantly higher levels than Allo (*P* < 0.01) and Str (*P* < 0.04).

## 4. Discussion

Scaffolds have been widely used for maxillofacial bone regeneration [33, 34]. Although composite scaffolds that contain alginate/gelatin have been fabricated [7, 35], utilizing FDBA or SrHA to reinforce the hydrogel scaffold has not been analyzed previously. Our results showed that lower mechanical strength, degradation, and swelling ratio are expected in the case of strengthening scaffolds with *β*-TCP compared to using FDBA or its combination with SrHA. Moreover, FDBA or a combination of FDBA and SrHA provided a more suitable platform for rBMSCs proliferation and differentiation than those reinforced with *β*-TCP. Besides, using SrHA, the proliferation of rBMSCs was not altered significantly, while their osteogenic differentiation improved.

To improve the stability of the gelatin, alginate has been used in this study. Although the biocompatibility of this hydrogel has been shown previously, there are still concerns about its complete degradation since mammalian macrophages do not have the required enzymes for this procedure [36]. Thus, this study has tried to minimize the amount of the utilized alginate to prevent the scaffold's collapse. In this study, only 2.5% of the scaffolds are composed of alginate, which is significantly lower than in similar articles [8, 27, 37].

Most studies that printed hydrogel scaffolds freeze-dried them to increase their mechanical stability [27]. Based on previous studies, Zn_2_SiO_4_ has been identified as the mineral phase incorporated into the alginate/gelatin nanocomposite hydrogels. This study has shown that the inclusion of Zn_2_SiO_4_ enhances the stability, and mechanical properties, and reduces weight loss by decreasing the swelling ratio. The findings of this study align with our research, confirming the beneficial effects of incorporating the mineral phase in the hydrogels (11). In addition, it has been claimed that microporosities may be created in this procedure, which can improve the biological behavior of the scaffolds as well [38]. However, this procedure can alter the dimensions of the scaffold. Considering that one of the rationales of CAD-CAM technology is the fabrication of a site-specific scaffold, freeze-drying can impair the marginal fit of the scaffolds, which is a crucial component for tissue integration of the scaffolds. In addition, freeze-drying complicates the fabrication procedure, increases the costs, and adversely impacts the biological agents that were used in the scaffold.

Eliminating freeze-drying from the fabrication procedure has decreased the mechanical strength of the scaffolds of this study. Meanwhile, studies that did not freeze-dry their hydrogel scaffolds reported comparable results to ours, and even our outcomes were higher in some instances [39–41]. However, it has to be considered that mechanical strength is significantly impacted by both the composition and design of the scaffolds. In addition, the cross-linking method can also affect the mechanical strength. Ye et al. [42] cross-linked their samples with glutaraldehyde and reported 1–10 MPa of compressive modulus. Although it was significantly higher than our results, considering the biological drawbacks of glutaraldehyde was not considered in our paper [43]. Increasing the amount of alginate is another way to improve the mechanical strength of the hydrogel scaffolds [39]. However, considering its possible biological drawbacks, only 2.5% of the scaffolds fabricated in this study consist of alginate.

FDBA and *β*-TCP were the calcium phosphate-derived bioceramics used in this study as the mineral phase of the scaffold. Although *β*-TCP has been widely used to reinforce synthetic polymers, specifically PCL and PLA [27, 44, 45], limited studies have analyzed hydrogel scaffolds reinforced with these materials. Motamedian et al. [46] performed a study to compare the behavior of stem cells seeded on *β*-TCP and FDBA and showed that although the former provides a better platform for cell proliferation, cells seeded on FDBA showed higher levels of osteogenic differentiation. Our results showed that scaffolds reinforced with FDBA had better outcomes concerning cell proliferation and osteogenic differentiation than those reinforced with *β*-TCP. However, limited studies have compared the behavior of stem cells seeded on FDBA and *β*-TCP, and more articles are required on this concern.

The ratio of the utilized material in the printing blend can impact the precision of the fabrication procedure. Giuseppe et al. [39] performed a study to analyze the impact of different wt % of alginate or gelatin on the accuracy of the fabrication procedure. It has been shown that increasing the gelatin weight ratio to more than 8% of the blend can decrease the precision, and there is no significant difference between the results of scaffolds containing 6% or 8% gelatin. Scaffolds with 7.5% gelatin content were fabricated in the current study and showed good printing accuracy.

In this study, 50% of the fabricated scaffolds consisted of bioceramics content. Since the main aim is to fabricate scaffolds with maximum compositional resemblance to the natural bone, the bioceramic content has to be increased to significantly higher levels than the mentioned value [47]. However, increasing that is accompanied by a more complicated fabrication procedure since it increases the possibility of nozzle clogging [48–50]. Besides, with higher percentages of bioceramics, scaffolds are prone to higher degradation rates and lower mechanical strength [14]. As scaffold stability in the initial phases of bone regeneration is a crucial step for successful treatment, a careless increase in the ceramic content can adversely impact the treatment's success. Conversely, bioceramics increase surface roughness and hydrophilicity, improving the scaffold's biological features [51].

It is well known that a 3D-printed interconnected porous microstructure is crucial for capillary penetration, cell-cell communication, and diffusion of nutrients [52]. Hassan et al. [53] reported in their systematic review that scaffolds with a pore diameter of around 400 *μ*m and a total porosity of >50%. This structure supported cell infiltration and attatchment as illustrated in SEM images.

### 4.1. Limitations

This study was limited in that different wt % of SrHA, *β*-TCP, and FDBA could not be evaluated to optimize their percentages in the scaffolds *in vitro*. Moreover, the *in vivo* experiment was not conducted, but it can provide outcomes with a higher level of evidence concerning the superiority of one of the scaffolds to the others. Moreover, studies tend to fabricate bioprinted scaffolds with somehow evenly distributed cells. However, since this procedure requires a more complicated and expensive fabrication setup, stem cells were cultivated on the scaffolds according to the traditional models. Meanwhile, it is worth mentioning that there is no strong evidence related to the superiority of the bioprinting procedure compared to conventional cell seeding.

## 5. Conclusion

It can be concluded that to improve the biological and physicochemical behavior of gelatin/alginate hydrogel scaffolds, FDBA and its combination with SrHA can be considered as better options compared to *β*-TCP to improve the cell proliferation, osteogenic differentiation, degradation, swelling ratio, and mechanical strength of gelatin/alginate scaffolds. Besides, adding SrHA to FDBA can enhance the osteogenic differentiation of stem cells while having no significant impact on stem cell proliferation.

## Figures and Tables

**Table 1:** Weight ratio of the utilized materials in study groups.

Study group	Compositions (%)		
	Bioceramic (w/w %)	Alginate (w/v %)	Gelatin (w/v %)
TCP	*β*-TCP (50%)	7.5	2.5
Allo	FDBA (50%)	7.5	2.5
Str	FDBA (37.5%) + SrHA (12.5%)	7.5	2.5

## Data Availability

The data used to support the findings of this study are available from the corresponding author upon request.
